# Stratification of Prognosis of Triple-Negative Breast Cancer Patients Using Combinatorial Biomarkers

**DOI:** 10.1371/journal.pone.0149661

**Published:** 2016-03-01

**Authors:** Yong Yue, Kristine Astvatsaturyan, Xiaojiang Cui, Xiao Zhang, Benedick Fraass, Shikha Bose

**Affiliations:** 1 Department of Radiation Oncology, Cedars-Sinai Medical Center, Los Angeles, California, United States of America; 2 Department of Pathology, Cedars-Sinai Medical Center, Los Angeles, California, United States of America; 3 Departments of Surgery, Cedars-Sinai Medical Center, Los Angeles, CA, United States of America; 4 Biostatistics and Bioinformatics Core, Cedars-Sinai Medical Center, Los Angeles, California, United States of America; Georgetown University, UNITED STATES

## Abstract

**Background:**

Triple-negative breast cancer (TNBC) is highly diverse group of cancers, and generally considered an aggressive disease associated with poor survival. Stratification of TNBC is highly desired for both prognosis and treatment decisions to identify patients who may benefit from less aggressive therapy.

**Methods:**

This study retrieved 192 consecutive non-metastasis TNBC patients who had undergone a resection of a primary tumor from 2008 to 2012. All samples were negative for ER, PR, and HER2/neu. Disease-free-survival (DFS) and overall-survival (OS) were evaluated for expression of immunohistochemical biomarkers (P53, Ki-67, CK5/6 and EGFR), as well as clinicopathological variables including age, tumor size, grade, lymph node status, pathologic tumor and nodal stages. The cutoff values of the basal biomarkers, EGFR and CK5/6, were estimated by time-dependent ROC curves. The prognostic values of combinatorial variables were identified by univariate and multivariate Cox analysis. Patients were stratified into different risk groups based on expression status of identified prognostic variables.

**Results:**

Median age was 57 years (range, 28–92 years). Patients’ tumor stage and nodal stage were significantly associated with OS and DFS. EGFR and CK5/6 were significant prognostic variables at cutoff points of 15% (p = 0.001, AUC = 0.723), and 50% (p = 0.006, AUC = 0.675), respectively. Multivariate Cox analysis identified five significant variables: EGFR (p = 0.016), CK5/6 (p = 0.018), Ki-67 (p = 0.048), tumor stage (p = 0.010), and nodal stage (p = 0.003). Patients were stratified into low basal (EGFR≤15% and CK5/6≤50%) and high basal (EGFR>15% and/or CK5/6>50%) expression groups. In the low basal expression group, patients with low expressions of Ki-67, low tumor and nodal stage had significantly better survival than those with high expressions/stages of three variables, log-rank p = 0.015 (100% vs 68% at 50 months). In the high basal expression group, patient with high basal expression of both biomarkers (EGFR >15% and CK5/6 >50%) had worse survival (mean DFS = 25 months, 41.7% event rate) than those patient with high expression of either one marker (mean DFS = 34 months, 25.5% event rate).

**Conclusions:**

Immunoexpression of basal biomarkers, EGFR and CK5/6, is useful in predicting survival of TNBC patients. Integrated with Ki-67, tumor and nodal stages, combinatorial biomarker analysis provides a feasible clinical solution to stratify patient risks and help clinical decision-making with respect to selecting the appropriate therapies for individual patients.

## 1. Introduction

Triple-negative breast cancer (TNBC), characterized by absence of expression of estrogen and progesterone receptors (ER, PR), and human epidermal growth factor receptor 2 (HER2), has been shown to be molecularly heterogeneous in gene expression analyses [1,2]. The majority of TNBC is with aggressive clinical course, higher rate of metastases, and shorter survival, thus requiring aggressive multidisciplinary treatment with surgery, radiation and chemotherapy [3,4,5]. The remaining TNBCs are less aggressive, have a more favorable prognosis, and may benefit from certain hormonal or targeted therapies.

Currently, the diagnosis and treatment for TNBC subtypes are not differentiated. Stratification of TNBC prognosis would be highly desirable to identify patient who may be spared from aggressive therapy. Gene profiling stratification would be a direct approach to this need, however classifying a single cancer into a gene expression subtype is impracticable in clinical practice, particularly for screening specific targeted genetic events which are occur at relatively low frequency [6–8]. The most widely accepted method in clinical practice is through identification of significant immunohistochemistry (IHC) surrogates for basal-like breast cancer, e.g. epidermal growth factor receptor (EGFR) and basal cytokeratins (CK5/6). However, prognostic stratification relying on individual biomarkers is prone to lack of standardization in patient risk management due to the heterogeneity in the staining and the absence of defined cutoffs of IHC based surrogates. Viale *et al*. [9] reported that immunoreacitvity of EGFR may have different prognostic values at different cutoff points, and low expression of basal biomarkers may not necessarily lead to poor survival. The survival prediction in clinical practice becomes incrementally challenged when the immunoreactivities of basal biomarkers are considered in concert with other clinicopathological variables [10–12]. These complicated clinical interactions often lead to difficulties in determining appropriate treatment [13]. A systematic survival prediction approach based on the integrative clinicopathological information would be highly desired to improve patient risk stratification and targeting of treatment, and further facilitate breast cancer management in routine clinical practice.

The goal of this study is to assess the prognostic value of combinatorial biomarkers in stratifying the risks of TNBC patients. In our approach, we first evaluate the individual prognostic value of basal biomarkers and other clinical variables using univariate Cox analysis through association with the survival outcomes [14]. In particular, we examine the prognostic value of basal biomarker EGFR and CK5/6 at different cutoff points, and estimate the optimal cutoff point with association to time-dependent survival outcome [15–18]. Based on the significant biomarkers/variables, the prognostic contributions of combinatorial biomarkers are identified by multivariate Cox analysis, and further used to stratify patient risks. Since clinicopathological data can be acquired during diagnosis, the proposed method could facilitate screening patients with good prognosis for less aggressive therapy.

## 2. Materials and Methods

### 2.1. Patients

Information was retrospectively collected on 192 consecutive non-metastatic triple-negative breast cancer patients who had undergone a resection of primary tumors from 2008 to 2012. This study was conducted in accordance with the Declaration of Helsinki, and approved by the Institutional Review Board of Cedars-Sinai Medical Center. No consent was needed in the study, and patient records/information was anonymized and de-identified prior to analysis. All patients were histologically confirmed as invasive carcinoma, and all patients received radical mastectomy, modified radical mastectomy, or lumpectomy as primary treatment. Among them, 159 of these patients were treated with chemotherapy and/or radiotherapy. In addition, 72 patients were treated with modified radical mastectomy (MRM), 64 patients were treated with breast conserving therapy (lumpectomy + radiotherapy, BCT), and 56 patients were received non-MRM/BCT treatments, e.g. mastectomy with radiotherapy, lumpectomy without radiotherapy. All tissue samples were examined by immunohistochemistry, and all samples with negative status of ER, PR, and HER2/neu were included.

### 2.2. Immunohistochemistry

Formalin-fixed, paraffin-embedded tissue sections were selected to include representative sections of carcinoma and adjacent normal breast tissue. All IHC stains were performed using a Polymer and/or SA-HRP Detection System. Estrogen (ER) and progesterone receptor (PR) status, Ki-67, P53, HER2/neu labeling indices were determined with the SP1, 1E2, K-2, DO7, and 4B5 antibodies (Ventana Medical Systems, Tucson, AZ, USA), respectively. ER, PR, Ki-67 and P53 immunoexpression was evaluated as the percentage of cells exhibiting definite nuclear staining. Cell proliferation (Ki-67) was assessed by counting at least 500 tumor cells (depending upon the availability of tumor). P53 was evaluated by counting at least 8–10 tumor cells in high power field. The slides were scored by the percentage of positive cells versus the total number of cells regardless of the staining intensity. In our clinical evaluation, the expression level of Ki-67 is considered low if the percentage of nuclear staining is less than 11%, intermediate if between 11% and 20%, and high if greater than 20%. Similar, the expression level of P53 is considered negative if the nuclear stains are less than 1%, low if 1–50%, and high if greater than 50%. The threshold for the definition of TNBC was a lack (<1% positivity) of any ER and PR immunoreactivity and a score of 0 or 1+ for HER2/neu immunoexpression and absence of amplification by fluorescent in situ hybridization. Immunostaining for CK 5/6, and EGFR was performed using monoclonal antibodies D5/16 B4 for CK5/6 (Dako, Glostrup, Denmark); and 2-18C9 for EGFR (Dako, Glostrup, Denmark). Results were recorded as the percentage of invasive carcinoma cells showing cytoplasmic and/or cytoplasmic membrane immunoreactivity for the corresponding antigen and the intensity of staining. The basal-like phenotype is defined as triple-negative (ER, PR, and HER2 negative) and EGFR or CK5/6 positive [4]. All IHC readings were verified by two pathologists, independently.

### 2.3. Statistical analyses

Patient’s overall survival (OS) and disease-free survival (DFS) were used as the endpoint for survival analysis, and disease-free survival (DFS) was defined from the date of the primary treatment to the date of first local recurrence or distant metastasis or death [19]. Statistical analysis was performed using R software 3.1.1 (The R foundation for statistical computing, Vienna, Austria) and SPSS 17.0 statistical software (SPSS Inc, Chicago, IL, USA). The statistical difference between subgroups of patients treated with chemotherapy/radiotherapy and patients without adjuvant therapy was evaluated by the Kruskal–Wallis test [20]. Prognosis values were examined among clinicopathological factors, including age, tumor size, modified Bloom-Richardson (MBR) score, nuclear grade, tubule formation, mitosis, pathologic tumor stage (pT), pathologic nodal stage (pN), overall stage, ER, PR, HER2/neu, P53, Ki-67, CK5/6 and EGFR. The association of these variables with survival was analyzed using a univariate Cox proportional hazard regression analysis. The resultant significant variables were selected as candidate prognostic indicators.

The cutoff values of EGFR and CK5/6 were estimated by the time-dependent ROC curves [15–18]. ROC curve is a typical method for displaying sensitivity and specificity of diagnostic markers. If the events happened before the observation, the ROC analysis can be used to identify the optimal cutoff point of biomarker. However, most of disease survival outcomes are time dependent, and events may not happen at the time of observation. For such censored data, ROC analysis may fail to correctly evaluate the sensitivity and specificity. To resolve this issue, we used the time-dependent ROC curve which is designed to process the censored data in evaluation of the sensitivity and specificity of biomarkers. In our implementation, survivalROC in R 3.2.2 was used to identify the threshold value by taking into account the censored survival outcome. Further, we evaluated the survival difference of expression level dichotomized at the cutoff points using log-rank tests and Kaplan-Meier curves. The combinatorial factors were identified by multivariate Cox analysis [21]. The quality of the analysis was evaluated by Akaike’s information criterion (AIC) [22,23], which is a measure of the relative quality of statistical models for a given set of data. A lower AIC value suggests a better goodness of fit and less information loss. Based on the expression status of factors, patients in the cohort were into different risk groups, and statistical outcome difference between groups was evaluated by log-rank. A *p*-value of 0.05 was considered significant.

## 3. Results

All patients were women with a median age of 57 years (range, 28–92 years), and the median follow-up was 22 months (range, 2–54 months). 145 patients were CK5/6 positive, and 175 patients were EGFR positive. A total of 179 patients were identified as basal-like (any EGFR and/or CK5/6 positivity). Patient and tumor characteristics are summarized in Table 1. Majority of the patients in our TNBC cohort had high Modified Bloom Richardson pathologic (MBR) grade and high proliferative rate (Ki-67 >20%). A large percentage of patients (69%) were negative lymph node whereas 31% patients had 1–17 metastatic lymph nodes. The results of Kruskal–Wallis test show that there was no significant differences between the patients treated with chemo/radiation therapy and those without adjuvant therapy in terms of survival months, pathological tumor and nodal stages, expressions of EGFR, CK5/6 and Ki-67 (all p>0.10). We evaluated the clinicopathological difference between patients treated with different treatment (MRM, BCT, non-MRM/BCT). The mean DFS was 38, 35 months for patients treated with MRM and BCT, respectively. The results of the Kruskal-Wallis tests show that there was no significant difference between two treatment groups (MRM vs BCT) with respective to patient and tumor characteristics (all p>0.1). Further we compared patients treated with MRM or BCT (n = 136) with patients treated with non-MRM/BCT (n = 56). There was also no significant difference between subgroup patients in terms of clinicopathological variables (all p>0.1).

**Table 1:** Patients characteristics.

Variables	Number of Patients (Percentage %)	Univariate Cox analysis (OS)	
		HR (95%CI)	*p*-value
All patients	192 (100)		
Age		1.044 (1.01–1.079)	**0.011**
≤50	67 (35)		
>50	125 (65)		
Size		1.018(0.996–1.041)	0.114
≤20mm	97 (52)		
>20mm	88 (48)		
MBR		1.347(0.1821–9.963)	0.770
<3	21 (11)		
= 3	167 (89)		
Tubule grade		0.999 (0.980–1.013)	0.990
<3	3 (2)		
= 3	180 (98)		
Nuclear grade		0.827 (0.114–5.99)	0.851
<3	14 (8)		
= 3	169 (92)		
Mitosis grade		1.231 (0.441–3.437)	0.691
<3	41 (23)		
= 3	141 (77)		
Pathology stage group		8.481 (3.247–22.15)	**0.001**
<3	144 (87)		
≥3	21 (13)		
Pathology tumor stage, pT		4.012 (2.455–6.558)	**0.001**
<3	161 (90)		
≥3	17 (10)		
Pathology nodal stage, pN		3.564 (2.041–6.225)	**0.001**
<1	116 (69)		
≥1	53 (31)		
Ki-67		1.005 (0.982–1.027)	0.681
≤50%	91 (47)		
>50%	101 (53)		
P53		1.006 (0.986–1.026)	0.569
≤50%	65 (46)		
>50%	77 (54)		
EGFR		1.001 (0.987–1.016)	0.857
= 0%	15 (8)		
0–15%	38 (20)		
>15%	137 (72)		
CK5/6		1.002 (0.985–1.018)	0.829
= 0%	47 (25)		
0–50%	106 (55)		
>50%	39 (20)		

**Fig 1:**
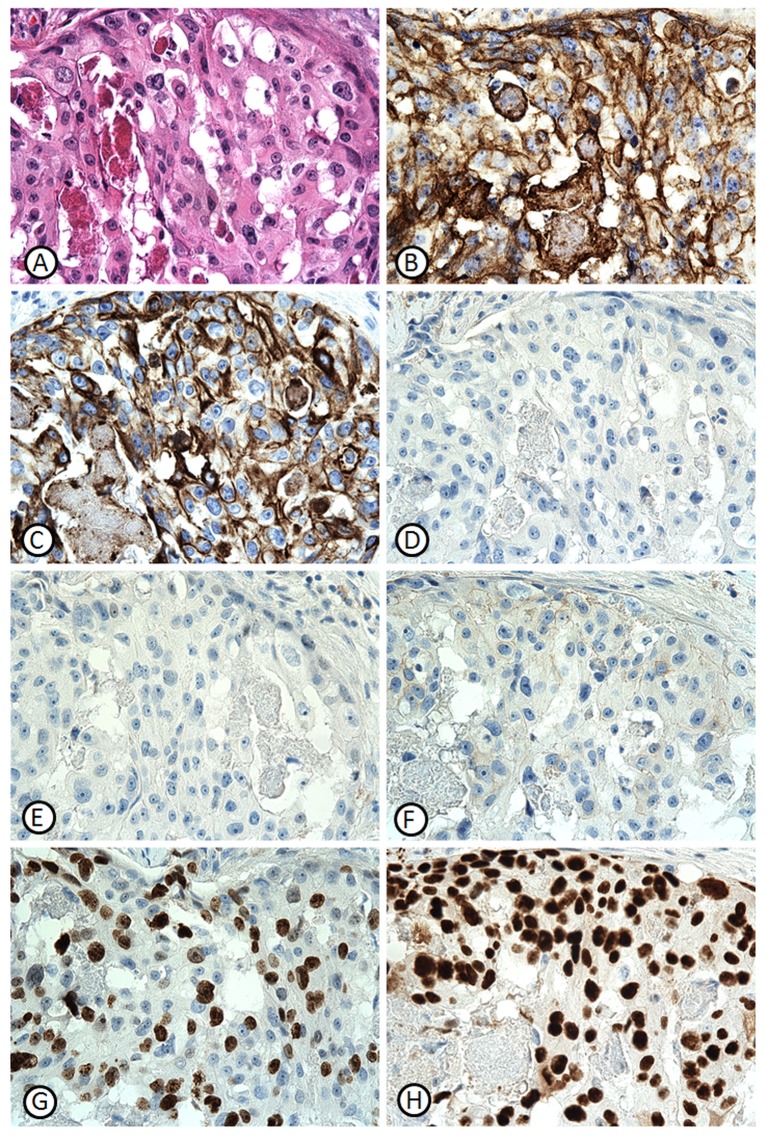
Immunoreactivity of 69-yr-old patient with T1bN0M0 TNBC. (A) Hematoxylin and Eosin (H&E), (B) EGFR (>70%), (C) CK5/6 (>70%), (D) ER, (E) PR, (F) HER2, (G) Ki-67 (>60%), and (H) P53 (>90%). The patient was undergone BCT, had 3.8 event free survival and 22.8 months overall survival.

Fig 1 illustrates the immunoreactivity of a 69-yr-old TNBC patient with low clinical pathological stage (T1bN0M0) and high expression of EGFR (>70%), CK5/6 (>70%), Ki-67 (>60%), and P53 (>90%). The patient was undergone breast conserving therapy (BCT), had 3.8 event free survival and 22.8 months overall survival.

### 3.1. Results of univariate Cox analysis

We first examined the significance of clinicopathological variables using univariate Cox analysis. As shown in Table 1, age was significantly associated with OS (p = 0.011), and clinical tumor stage, nodal stage and overall stage were also significance (p = 0.001, 0.001, and 0.001, respectively). Further, clinical tumor stage, nodal stage and overall stage were also significantly associated with DFS (p = 0.001, 0.001, and 0.002, respectively, Table 2). Tumor size, MBR grade, Ki-67 and P53 were not significant in the univariate Cox analysis. The proliferation marker Ki-67 was highly expressed in our data with a median value of 51%. Although Ki-67 did not appear to be significant in univariate cox analysis, it had significant correlation to MBR grade (p<<0.0001), especially nuclear and mitotic grade (0.0009, <<0.0001).

**Table 2:** Prognostic value of clinicopathological variables in predicting disease free survival using univariate and multivariate Cox model.

Variables	Univariate Cox Analysis (DFS)		Multivariate Cox Analysis (DFS)	
	HR (95%CI)	*p*-value	HR (95%CI)	*p*-value
Age	1.020 (0.999–1.038)	**0.061**	-	-
Pathology, tumor stage, pT	1.877 (1.281–2.75)	**0.001**	5.104 (1.468–17.748)	**0.0103**
Pathology, nodal stage, pN	1.728 (1.231–2.425)	**0.001**	3.389 (1.511–7.601)	**0.0031**
Pathology, Stage Group	1.988 (1.272–3.106)	**0.002**	-	-
EGFR %, continuous	1.012 (1.002–1.022)	**0.021**	-	-
≤15% vs > 15%	4.066 (1.607–10.29)	**0.003**	6.124(1.400–26.786)	**0.0161**
CK5/6%, continuous	1.01 (1.001–1.019)	**0.034**	-	-
≤ 50% vs > 50%	2.308 (1.248–4.27)	**0.008**	2.678 (1.187–6.044)	**0.0177**
P53%, continuous	0.994 (0.990–1.010)	0.902	-	-
≤ 50% vs > 50%	1.028 (0.513–2.061)	0.938	-	-
Ki-67%, continuous	1.011 (0.990–1.024)	0.122	-	-
≤50% vs >50%	1.503 (0.834–2.707)	0.175	2.367(1.005–5.574)	**0.0487**

**Fig 2:**
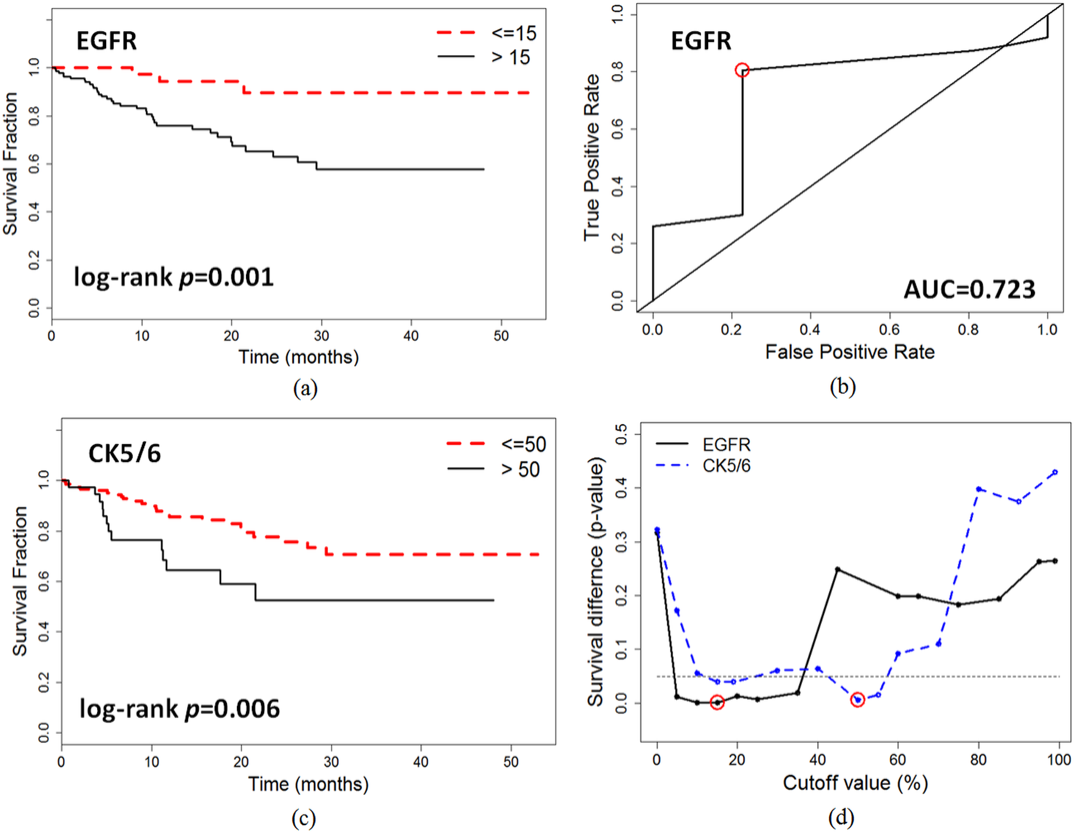
Kaplan Meier curves of disease free survival for basal biomarkers. (a) EGFR at cut-off level 15% with log-rank p = 0.0016, (b) time-dependent ROC analysis of EGFR with AUC = 0.723, where cutoff point 15% (red circled), (c) CK5/6 at cut-off level 50% with log-rank p = 0.0066, and (d) the significance of survival difference at different cutoff values for EGFR and CK5/6. The most significant cutoff values were red-circled with 15% for EGFR and 50% for CK5/6.

EGFR expression was significantly associated with DFS in both continuous (p = 0.019) and discrete status (p = 0.005). The cutoff value of EGFR was estimated by time-dependent survival ROC analysis. As a result, the cutoff value 15% maximized both sensitivity and specificity of the survival outcome with AUC = 0.723 (Fig 2(B)). As shown in Kaplan-Meier curve (Fig 2(A)), patients were stratified into two risk groups with EGFR (≤15% vs >15%) with log-rank p = 0.0016. The patients with high EGFR (n = 137) had worse survival outcome than those with low EGFR (n = 53). We also examined the different cutoff percentiles (0–99%) using log-rank tests and Cox regression analysis. The p-values for thresholds 1, 15 and 50% were 0.202, 0.001 and 0.322 for log-rank tests, and 0.347, 0.005, 0.060 for Cox analysis, respectively. The significance of survival difference at different cutoff values for EGFR is shown in Fig 2(D), where the most significant cutoff values were red-circled at 15% of EGFR. The results indicate that the low expression (such as 1%) of EGFR does not necessarily correlate with poor survival outcome. On increasing cutoff values, the classification became significant from 5% to 30%, and then lost significance at higher percentages.

CK5/6 was significant associated with DFS in continuous (p = 0.034) and discrete status (>50 vs ≤50, p = 0.008). The chi-square tests show that CK5/6 was significantly correlated to MBR (*p* = 0.009), tumor mitotic index (*p* = 0.007), EGFR (*p* = 0.003), and P53 (p<0.001). The time-dependent ROC analysis suggested that 50% was the optimal cutoff point for CK5/6 with AUC = 0.675. The Kaplan-Meier curves of the cut-off value are shown in Fig 2(C), and the significance of survival difference at different cutoff is shown in Fig 2(D). The same cut-off value also has been used in other studies [11].

### 3.2. Identification of prognostic combinatorial biomarkers

We performed a multivariate Cox regression analysis to assess the prognostic value of combinatorial biomarkers for disease-free survival. The candidate variables of the multivariate analysis were obtained from significant univariate variables as well as those used in the literature [16, 21]. As shown in Table 2, multivariate Cox analysis identified five significant prognostic factors, including pathology tumor stage (*p* = 0.0103), nodal stage (*p* = 0.0031), EGFR (*p* = 0.0161), CK5/6 (p = 0.0177), and Ki-67 (p = 0.0468). We further examined the quality of the multivariate analysis using the Akaike’s information criterion. A lower value of AIC signifies a superior model with a better goodness fit and less information loss. If only two clinical variables (tumor stage, and nodal stage) were used in multivariate analysis, the performance of analysis was worse (AIC = 229.5). When basal biomarkers (EGFR and CK5/6) were included, the performance was significantly improved with AIC = 216.1. Finally, Ki-67 further improved the robustness of multivariate analysis resulting in optimized five-variable analysis with AIC = 213.8.

### 3.3. Patient risk analysis

With the integration of prognostic clinical and IHC biomarkers, patients were stratified into different risk-groups based on expression status of 5 prognostic variables identified by multivariate analysis. Basal biomarkers, EGFR and CK5/6, were used as the primary classification variables to stratify patients into a low basal (EGFR≤15% and CK5/6≤50%) and high basal (EGFR>15% and/or CK5/6>50%) risk groups. In the low basal risk group, patients were further stratified into two subgroups based on status of other three prognostic variables, pT, pN and Ki-67. Specifically, patients in risk group 1 (n = 20) had low expression of Ki-67(≤50%), and low clinical tumor stage (≤3) and nodal stage (≤1), whereas risk group 2 (n = 30) included any patient with high expressions/values of Ki-67, pT, or pN. In the high basal risk group, patients with single high expression (EGFR >15% or CK5/6 >50%) were classified into risk group 3 (n = 106), likewise patients with double high expression of basal biomarkers (EGFR >15% and CK5/6 >50%) were in group 4 (n = 36).

**Fig 3:**
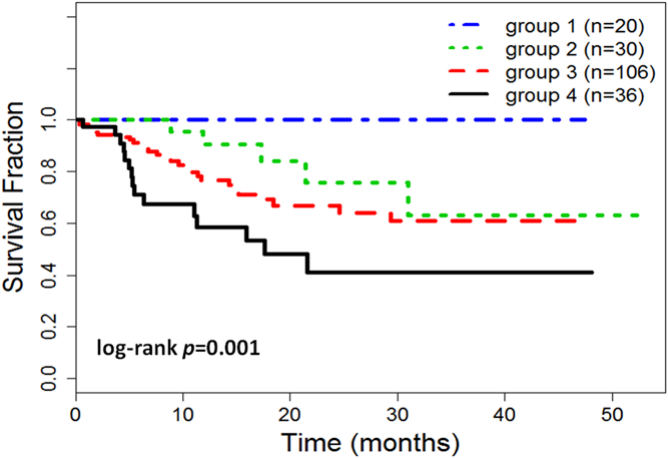
Kaplan Meier curves of disease free survival of risk groups for TNBC patients. The patients were stratified into different risk groups. Two low basal (EGFR≤15% and CK5/6≤50%) risk groups: group 1 (n = 20) with low expressions/values of EGFR (≤15%), CK5/6 (≤50%), Ki-67 (≤50%), pT (≤ 3), and pN (≤ 1), and group 2 (n = 30) with low expressions of EGFR and CK5/6, and any high expressions/values of Ki-67, pT, and pN. Two high basal risk groups: group 3 (n = 106) with single high basal expression (EGFR>15% or CK5/6>50%), and group 4 (n = 36) with double high basal expressions (EGFR>15% and CK5/6>50%).

Fig 3 shows the risk stratification of TNBC patients with log-rank p = 0.001. The results of risk groups with median values of prognostic variables are listed in Table 3. The results show that 10.4% of 192 TNBC patients were in the risk group 1 with a mean DFS of 48 months. The risk group 2 had a mean DFS of 37 months, and 16.6% patients of this group had one or multiple events whereas patients in group 1 had zero event. Patients in group 1 had a significantly better outcome than those in group 2, log-rank p = 0.015 (100% vs 68% at 50 months). For the high basal groups, the majority of TNBC patients (55.2%) were in group 3 with a mean DFS of 34 months. The risk group 4 had the shortest mean DFS (25 months), highest median value of EGFR (70%) and CK5/6 (77.5%). There were 41.7% patients in group 4 suffered to one or multiple events, which had the highest event rate compared to other risk groups.

**Table 3:** TNBC patient risk groups stratified by EGFR, CK5/6, Ki-67, pT and pN.

Risk Groups	Patient Number (%)	Mean DFS Months	Events in the Group (%)	Median EGFR (%)	Median CK5/6(%)	Median Ki-67 (%)	Median tumor stage	Median nodal stage
1	20 (10.4%)	48	0 (0%)	2.5	3.0	31.5	T2	N0
2	30 (15.6%)	38	5 (16.7%)	5.0	3.0	66.0	T2	N1
3	106 (55.2%)	34	27 (25.5%)	60.0	5.0	50.0	T2	N0
4	36 (18.8%)	25	15 (41.7%)	70.0	77.5	56.0	T2	N0

## 4. Discussion

Triple negative breast cancer is highly diverse group of cancers, and generally considered an aggressive disease associated with poor survival. Recent gene expression microarray studies have revealed at least six subtypes. Some TNBC patients, e.g. luminal androgen receptor or molecular apocrine cancers, may have better prognosis than the remaining majority [3,4]. The selection of gene expression subtypes for a single cancer is challenging in the clinics [6–8]. An efficient approach would use immunohistochemistry surrogates and other available clinical information to stratify TNBC patients upfront of treatment. CK5/6 and EGFR have been widely accepted as biomarker for basal-like breast cancer. Viale *et al*. [9] and Zhang *et al*. [10] reported that EGFR immunoreactivity correlated significantly with worse prognosis in their TNBC patients. Thike *et al*. [11] reported that basal cytokeratins had significant prognostic values in their cohort of patients. In addition, proliferation biomarker Ki-67 is also reported to be significantly associated with a high histologic grade, and poor survival in TNBC patients [16]. In instances when the expression of these biomarkers is not in concert with each other, it is challenging to determine appropriate treatment based on an individual biomarker. From a cohort of 192 TNBC patients, this study identified five significant clinical variables/biomarkers, including pathological tumor stage, nodal stage, EGFR, CK5/6 and Ki-67. The approach presented here can be used to evaluate the prognosis of patients at diagnosis and help clinical decision-making with respect to selecting the appropriate therapies for individual patients.

The risk analysis of TNBC patients mainly relied on the expression status of basal biomarkers. As suggested in results of multivariate Cox analysis, patients with high expression of both EGFR and CK5/6 had the worst prognosis with 41.7% event rates. To improve survival outcome, these patients could be administrated multidisciplinary treatment strategies, e.g. surgery, radiation and chemotherapy. Our results also indicate that patients with low expression of both basal biomarkers may not necessary have good prognosis. Three additional prognostic variables (Ki-67, tumor and nodal stages) were important for further stratifying risk in these patients. The risks of patients with low basal expression, high values of Ki-67, tumor or nodal stage (group 2) were closed to the risks of patients in group 3 (as shown in Fig 3), who had high expression of either one of the basal biomarker. Only those patients with low clinical tumor and nodal stages and low expression of EGFR, CK5/6 and Ki-67 had better prognosis. The percentage of such low-risk patients was 10.4% of total TNBC patient population, and 40% of the patient with low basal expressions. These patients could be candidates for being managed with less aggressive treatment strategies.

Proliferation marker Ki-67 is an important variable for patient survival [16]. High Ki-67 is associated with a higher histologic grade, larger tumor size, presence of axillary lymph node metastasis, and worse outcome. Since TNBCs typically exhibit high tumor grade and high proliferation rate, the expression of Ki-67 is usually high in most TNBCs. For our study, the median value was 51%. Although Ki-67 did not appear to be significant in univariate cox analysis, it had significant correlation to MBR grade. Further analysis showed that Ki-67 improved the performance of multivariate Cox analysis by reducing AIC value. Our results of risk analysis indicate that Ki-67 may play different roles in survival outcome. For the low basal expression, high Ki-67 may indicate a worse survival (e.g. risk group 2 in Table 2). However, for high basal expression, the contribution of Ki-67 expression to survival became trivial (e.g. risk group 3 and 4). This may rationally relate to EGFR signaling, which also regulates cell proliferation and differentiation. Tumor cells with low Ki-67 but high EGFR expression would still exhibit high proliferation activities, and lead to a worse survival outcome.

Although immunoreactivity of biomarker should be considered as a biological continuum, setting a threshold can provide clinical benefit by identifying those patients at high risk. Several methods have been proposed to estimate an optimal cutoff point of biomarkers. ROC curve analysis is a widely used method to select biologically or clinically relevant threshold for IHC tumor positivity [24]. However, the method may be suboptimal since censored data are not treated adequately in the ROC analysis. Other studies [9,25] have also used the maximum log-rank statistical value to find an empirical threshold among each possible cutoff points, which may lead to local minimum due to lacking theoretical rigorousness. In this study, we used time-dependent ROC curves to determine the optimal cutoff values [15–18], and further used log-rank tests and Kaplan-Meier curves to verify the statistical significance of those thresholds. Our results suggest that identified cutoff points of EGFR and CK5/6 have significant detrimental prognostic effect on basal-like TNBC. Despite the statistically significant detrimental prognostic effect associated with combinatorial biomarkers, a potential bias still exists due to the retrospective nature of the study and relative short follow-up time. Further studies using a larger independent data set and longer follow-up time will be necessary to confirm the prognostic value of the proposed combinatorial pathological variables before implementation in clinical practice.

## 5. Conclusions

Our study identified five prognostic clinicopathological variables in predicting survival of TNBC patients. Quantitative analysis of cutoff values of immunoreactivity for basal markers EGFR and CK5/6 improves the accuracy of stratification of survival outcome. Integration with Ki-67, tumor and nodal stage, combinatorial biomarker analysis provides a feasible clinical solution to stratify TNBC patient risks and help clinical decision-making with respect to selecting the appropriate therapies for individual patients. Further studies using a large data set will be necessary to confirm the prognostic value of the extent of the combinatorial variable in stratification of patient risks.

## Abbreviations

TNBC: triple-negative breast cancer
IHC: immunohistochemistry
ER: estrogen receptor
PR: progesterone receptor
HER2: human epidermal growth factor receptor 2
EGFR: epidermal growth factor receptor
CK5/6: Cytokeratin 5/6
ROC curve: Receiver Operating Characteristic curve
